# Self-determination Theory as a Lens to Explore the Implementation Challenges of Telehealth

**DOI:** 10.5195/ijt.2024.6648

**Published:** 2025-01-15

**Authors:** Yafit Gilboa, Michal Pagis, Kathleen Lyons

**Affiliations:** 1 School of Occupational Therapy, The Hebrew University of Jerusalem, Jerusalem Israel; 2 Department of Sociology and Anthropology, Bar-Ilan University, Israel; 3 Occupational Therapy, MGH Institute of Health Professions, Boston, MA, USA

**Keywords:** Autonomy, Competence, Motivation, Relatedness, Remote rehabilitation

## Abstract

Many benefits can be gained from telehealth, including reduced travel time, flexible work schedules, increased patient satisfaction, and a cost-effective method of providing care. Furthermore, telehealth provides rehabilitation professionals with the opportunity to observe people in their natural environment as they conduct their daily activities and identify any barriers to their functioning. Despite these advantages, and after a substantial amount of research supporting its effectiveness, telehealth remains relatively underutilized.

Self-determination theory (SDT) is a theoretical framework for explaining motivation in terms of three basic psychological needs: competence, relatedness, and autonomy. Using the SDT, we suggest analyzing the motivational challenges faced by the therapists when implementing telehealth.

We assert that the transition to a remote treatment model can be advantageous for rehabilitation professionals since it provides them with a greater degree of autonomy. Nevertheless, a turning point can only be achieved if relatedness and competence are maintained.

## Telehealth: An Accepted Method of Rehabilitation Delivery

Rehabilitation as defined by the World Health Organization is a set of person-centered services designed to reduce disability and maximize a person's ability to function within their home and community environments (WHO, 2024). Rehabilitation specialists (e.g., occupational therapists, physical therapists, speech-language pathologists, nurses, psychologists, and physiatrists) work with people with physical, mental, and developmental disorders in various settings including hospitals, schools, clinics, and community settings. Within the past two decades, telehealth guidelines have been developed within rehabilitation (Peretti et al., 2017).

Over the past two decades, telehealth has proven to be a feasible, safe, and acceptable service delivery model to improve access and support the continuity of care for individuals of all ages with a wide range of health conditions in many settings (Abbott-Gaffney et al., 2022; Baffert et al., 2023; Brigo et al., 2022). Multiple studies have also demonstrated effectiveness in varying populations, including children with autism (Little et al., 2018), adolescents with myelomeningocele (Steinhart et al., 2021), cancer survivors with cognitive decline (Maeir et al., 2023), adults with acquired brain injuries (Beit Yosef, Jacobs, et al., 2022), and older adults after hip fracture (Gilboa et al., 2019). Moreover, recently published systematic reviews concluded that telehealth is a practical alternative to conventional in-person therapy services, which may have equivalent or even better outcomes than in-person interventions (Knepley et al., 2021; Pang et al., 2023).

Despite the compelling advantages of this service delivery model, it is underutilized in clinical practice (Almog & Gilboa, 2022; Ninnis et al., 2019; Stampa et al., 2024). As alternative service delivery models were urgently needed during COVID-19, many rehabilitation centers have not used implementation strategies or a systematic approach to their implementation of telehealth (Stampa et al., 2024).

Studies have suggested that the well-documented gap between telehealth research and practice is the result of several interacting factors, including limited time and resources of practitioners, insufficient training, lack of feedback and incentives, and inadequate organizational infrastructure and systems to support implementation (Abbott-Gaffney et al., 2022; Ben Zagmi et al., 2021; Glasgow et al., 2003; Kringle et al., 2023; Loubani & Rand, 2023). Specifically, there is a general consensus that the success of innovations becoming part of existing and new clinical routines relies mainly on clinicians’ acceptance, adoption, and sustained compliance (Beit Yosef, Maeir, et al., 2022). There may also be hesitation to embrace this changing delivery model (Almog & Gilboa, 2022).

## Telehealth and Professional Motivation and Identities

Studies of telehealth examined care workers' receptivity towards remote health services provided through different digital platforms and applications. The main theoretical models used to examine receptivity are technology acceptance models. These emphasize features of the technology that interface with workers and organizations, such as the user-friendliness of the app, its usefulness, questions of digital media privacy, and availability of technical support (Garavand et al., 2022). Yet even as technology improves and becomes more friendly and intuitive, health-care workers still express hesitation that reduces receptivity (Chow et al., 2022; Keel et al., 2023). Thus, the nature of the technology and the technical support provided do not fully explain why rehabilitation professionals resist the use of telehealth. We suggest shifting from a focus on technology to a focus on work motivations that determine professional identities, and how these impact the willingness to shift to remote medicine.

**Figure 1:**
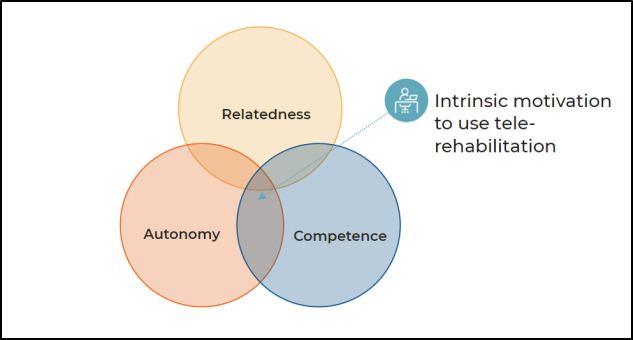
Telehealth According to the Self-determination Theory (SDT)

Self-determination theory (SDT) is a theoretical framework used to explain active engagement as a motivation to change behavior (Deci & Ryan, 2013). Fundamental to SDT is the idea that motivations are largely intrinsic and mediated by a set of basic psychological needs: competence, relatedness, and autonomy (see Figure 1). These basic psychological needs frame the development of identity (La Guardia, 2009) and are essential for psychological health and well-being, as they facilitate effective functioning in social settings (Deci & Ryan, 2013).

SDT was expanded to include research on work and organizational culture, specifically suggesting that both employees’ performance and their well-being are affected by the motivation they have for their job activities (Deci et al., 2017). A work design influenced by SDT enlivens these constituent identity processes through intrinsically and extrinsically motivated activity, based on the premise that work arrangements should enable employees to satisfy their basic psychological needs (La Guardia, 2009). The introduction of new technologies and work models, such as telehealth, may affect work design and may lead to changes in work motivation (Gagné et al., 2022).

Little research has been conducted to understand the impact of the implementation of telerehabilitation on the professional motivation and identity of rehabilitation professionals (Beit Yosef, Maeir, et al., 2022; Damhus et al., 2018). A review of the literature on remote working at large (including but not specific to the health professions) reveals both benefits and challenges to professional identities. Benefits include an increase in job autonomy and flexibility, which are related to higher job satisfaction, less work/family conflict, and reduced worker stress (Beckel & Fisher, 2022; Tavares, 2017). On the other hand, one of the challenges found in teleworking is professional isolation that tends to decrease relatedness (De Vries et al., 2019; Tavares, 2017), with workers reporting feeling lonely and missing in-person social communication. The negative effect on relatedness stems from the fact that interactions with patients and colleagues are a core concept of the professional identity of health-care professionals (Gagné et al., 2022). For example, in a study that examined the processes involved in implementing telehealth nursing services for patients at home with chronic obstructive pulmonary disease (COPD), the nurses reported that telehealth hindered them in establishing relationships with their patients, which negatively impacted their professional self-image and status (Hibbert et al., 2004). A recent study of mental-health professionals who shifted to teleworking found that when workers conducted video consultations, they missed personal details related to mood, gestures, and general body language (Kordelas, 2023).

As technological innovations change the nature of work, the third dimension, competence, can increase or decrease depending on the specific professional context. On one hand, information communication technology might satisfy competence needs by increasing access to global information and communication as well as the ability to analyze data (Gagné et al., 2022). At the same time, telerehabilitation introduces disruptions to long-standing models of expertise common to the professionals. Technology might thwart competence needs, as telerehabilitation removes in-person interaction it complicates nonverbal communication and disrupts procedures that rely on touch and physical co-presence. Therefore, rehabilitation practitioners must embrace a radically new mode of operation. They need to develop new capabilities for a medium in which embodied information is limited and is replaced by linguistic descriptions (Vestergaard, 2021).

In summary, the paradigm shift from in-person rehabilitation to telehealth transforms the nature of work for health professionals and therefore has organizational and occupational implications for therapeutic frameworks and the therapists (Creswell & Hirose, 2019). Understanding the impact of telehealth on the basic psychological needs for professional motivation and identities will enable us to develop a working model that maintains and enhances experiences of relatedness and competence.

We suggest that transitioning to a remote treatment model can be attractive for rehabilitation professionals because it provides more autonomy. However, it only serves as a turning point if it allows the maintenance of relatedness and competence. Key relevant barriers identified for the implementation of telehealth are technical issues and a lack of technical skills (Stampa et al., 2024). Factors that can improve the sense of competence include early socialization into telerehabilitation format and the technology. College and university rehabilitation programs should shift from a physical co-presence therapeutic model to one that incorporates remote digital technology use, at the early stages of education (Borges do Nascimento et al., 2023). In addition, hybrid venues that include remote service as an adjunct to traditional in-person contact can foster relatedness with patients and colleagues (Almog & Gilboa, 2022). Finally, rigorous implementation science regarding telerehabilitation is necessary and could be enhanced by the incorporation of Self-determination theory to inform development and testing of implementation strategies.
